# One-Stage Primary Total Knee Arthroplasty as the Treatment of Acute Septic Arthritis of the Native Osteoarthritic Knee: Report of 3 Cases and Review of Literature

**DOI:** 10.1016/j.artd.2025.101777

**Published:** 2025-07-28

**Authors:** Mohammad Mahdi Sarzaeem, Davood Feizi, Hamidreza Jamshidi Kouhsari, Farzad Amouzadeh Omrani, Ali Pourmojarab

**Affiliations:** Department of Orthopaedic Surgery and Traumatology, School of Medicine, Shahid Beheshti University of Medical Sciences, Tehran, Iran

**Keywords:** Septic arthritis, Total knee arthroplasty, Single-stage surgery, Osteoarthritis, Infection management, Joint replacement

## Abstract

We present three cases of acute septic arthritis in osteoarthritic knees managed with one-stage primary total knee arthroplasty and followed for 2 years. This approach, diverging from conventional staged protocols, combines thorough debridement, immediate biomechanical restoration, and extended antibiotic therapy. Patients exhibited significant symptomatic relief, functional recovery, and no recurrent infections. Literature supports the efficacy of single-stage revisions in chronic periprosthetic infections, yet its application to acute septic arthritis remains underexplored. Our findings highlight the importance of meticulous patient selection, considering host immunity, microbial virulence, and comorbidities. One-stage total knee arthroplasty offers a viable, streamlined alternative for well-selected patients, mitigating the morbidity of multiple surgeries and protracted recovery.

## Introduction

Acute septic arthritis of the knee, with mortality rates reaching 20% in elderly populations, constitutes a formidable clinical challenge necessitating prompt, coordinated intervention [[1], [2], [3]]. The rapid intra-articular proliferation of pathogens precipitates chondrocyte apoptosis and enzymatic cartilage degradation, culminating in irreversible joint destruction if inadequately addressed [4]. In patients with underlying osteoarthritis, the infectious insult intensifies degenerative deterioration, frequently mandating repetitive surgical interventions to restore joint integrity and alleviate incapacitating symptoms [5].

Conventional management encompasses emergent joint debridement, empirical broad-spectrum antibiotics, and protracted pathogen-specific therapy [4,6]. Yet, this approach may yield suboptimal functional outcomes in osteoarthritic patients, with many patients requiring late-stage salvage procedures such as arthrodesis or total joint arthroplasty [7]. The cumulative morbidity associated with multiple surgeries has catalyzed interest in one-stage primary total knee arthroplasty (TKA) as a definitive treatment strategy, integrating infection eradication and biomechanical restoration within a single surgical episode.

Despite theoretical advantages, concerns regarding reinfection risk, optimal antimicrobial stewardship, patient selection criteria, and long-term prosthetic survivorship persist [8,9]. In this report, we present three cases of acute septic arthritis superimposed on end-stage osteoarthritis successfully treated with one-stage primary TKA, underscoring the feasibility of this paradigm-shifting approach.

## Case histories

We report three patients diagnosed with acute septic arthritis superimposed on advanced osteoarthritis, all managed successfully with one-stage primary TKA. All patients in this case series provided explicit written informed consent authorizing the use of their anonymized clinical data for research purposes.

### Clinical presentation

#### Case 1

A 59-year-old male with well-controlled seropositive rheumatoid arthritis (RA) managed with methotrexate (15 mg/wk) and oral prednisolone (5 mg/d), as well as hypertension managed with losartan (160 mg/d), presented with a 10-day history of bilateral knee pain exacerbation, swelling, and low-grade fever. Body mass index (BMI) was 28.4 kg/m^2^; the American Society of Anesthesiologists (ASA) classification was *Physical Status II*. He was a nonsmoker and had a history of multiple intra-articular steroid injections, the most recent 2 months before admission. Physical examination revealed pronounced effusion, erythema, and a restricted range of motion, more severe on the right side. Synovial fluid analysis disclosed purulent effusion with leukocytosis (>100,000 white blood cells (WBCs)/mm^3^, 90% polymorphonuclear (PMN) predominance on the right side and 98,000 WBCs/mm^3^, 86% PMN on the left side), and cultures identified methicillin-sensitive *Staphylococcus aureus* (MSSA). The initial erythrocyte sedimentation rate (ESR) was 92 mm/h and C-reactive protein (CRP) was 185 mg/L.

#### Case 2

A 78-year-old male with non–insulin-dependent diabetes mellitus (hemoglobin A1c 7.2%), managed with metformin (1000 mg/d) and gliclazide (80 mg/d), presented with acute right knee pain, swelling, and impaired ambulation. BMI was 25.1 kg/m^2^; ASA classification was *Physical Status II*. He was a nonsmoker. He had received a 7-day course of IV antibiotics at an external facility but exhibited persistent symptoms. He reported an intra-articular injection of hyaluronic acid 4 weeks prior to admission. Examination elicited a ballotable patella indicative of an effusion and severe movement restriction. Aspiration revealed cloudy synovial fluid with 95,000 WBCs/mm^3^ (92% PMNs). Gram stain demonstrated gram-positive cocci in clusters; however, cultures from the aspirated fluid were negative. Intraoperative deep tissue cultures later grew MSSA. The ESR was 76 mm/h, and the CRP was 132 mg/L upon presentation.

#### Case 3

A 68-year-old female with ischemic heart disease (on aspirin, atorvastatin, and bisoprolol) and chronic obstructive pulmonary disease (on tiotropium inhaler), BMI 24.7 kg/m^2^, *ASA Physical Status II*, and an ex-smoker (quit 12 years ago), presented with fever (38.4°C), left knee swelling, and inability to bear weight. Three months prior, she had undergone a platelet-rich plasma injection in the affected knee. Aspiration yielded thick, purulent synovial fluid with 104,000 WBCs/mm^3^ (88% PMNs). Gram stain was positive for gram-positive cocci in clusters, and MSSA was isolated. ESR was 98 mm/h, and CRP was 201 mg/L.

**Figure 1:**
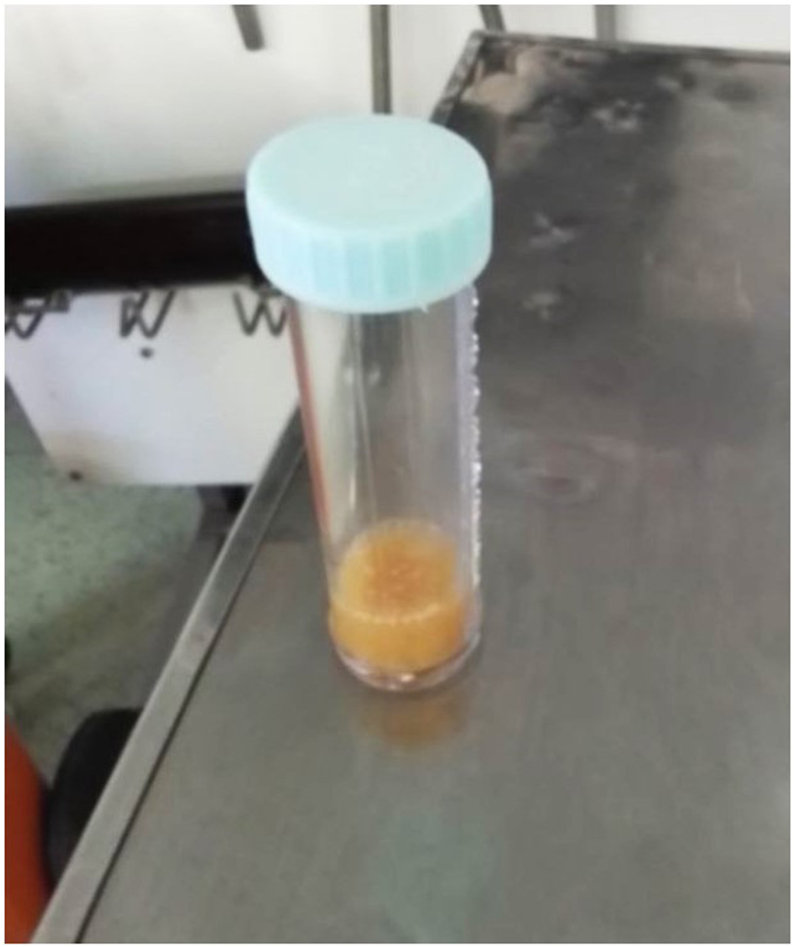
Grossly purulent synovial fluid aspirated from the patient’s knee joint, demonstrating the presence of a severe intra-articular infection. The cloudy, thick appearance is indicative of a high leukocyte count and bacterial proliferation, commonly seen in septic arthritis.

In all three cases, as described above, laboratory findings demonstrated leukocytosis, markedly elevated CRP and ESR, and purulent synovial aspirates (Fig. 1). Preoperative radiographs revealed end-stage bone-on-bone osteoarthritis with substantial joint space narrowing, osteophyte formation, and malalignments (since we routinely perform TKA using the kinematic alignment technique, preoperative three-joint alignment radiographs are not obtained as part of our standard protocol; therefore, the precise degree of preoperative varus or valgus malalignment is not determined).

### Surgical technique

In all cases, a standardized one-stage primary TKA protocol was implemented. In the first case, both knees were treated during the same surgical session using a bilateral approach. All TKA procedures in this series were performed manually, without robotic assistance or intramedullary canal instrumentation. Instead, we utilized a patented extramedullary distal femoral cutting block (US Patent No. 11,766,269 B2), developed by one of the authors (M.S.), which uses the readily accessible posterior and distal femoral condyles as reference surfaces for accurate alignment, obviating the need to violate the femoral canal [10]. This novel device, previously described and validated in clinical settings [11], allows precise perpendicular bone resection to the posterior femoral cortex and maintains compatibility with both mechanical and kinematic alignment strategies.

The surgical sequence began with a subvastus approach under spinal anesthesia and was followed by extensive synovectomy. Radical debridement was defined as the complete excision of all macroscopically infected synovium, necrotic tissue, purulent material, and any visibly eroded cartilage or subchondral bone. Following soft tissue debridement, the femoral bone cuts were performed using the patented distal femoral cutting system described above, without canal violation. The tibial cut was made using a standard extramedullary guide. Bone cuts were performed according to the kinematic alignment technique. A cruciate-retaining (CR) prosthesis was selected in all cases. This choice was based not only on the preservation of posterior cruciate ligament integrity in these patients but also on the implant philosophy of the lead surgeon, who routinely uses CR designs and kinematic alignment techniques as standard practice. Then the surgical team reassessed the field for residual infection. Once radical debridement was deemed complete, a pulsatile lavage using 3 liters of diluted povidone-iodine (0.35%) and an additional 3 liters of normal saline containing vancomycin (1 g/L) and gentamicin (80 mg/L) was performed. Then all instruments and suction tips were exchanged, the team re-gowned, and the operative field was re-prepped and redraped. Prosthesis implantation proceeded under strict aseptic precautions. The medullary canals were neither opened nor reamed at any stage.

Cemented antibiotic-loaded (Simplex P bone cement, Stryker, containing 1 g tobramycin) CR Triathlon primary prosthesis (Stryker, Kalamazoo, MI) were implanted, ensuring optimal fixation. No other intra-articular or local antibiotics were used beyond the tobramycin-loaded cement and the aforementioned irrigation protocol. In the first presented case, this procedure was repeated on the contralateral side in the same surgical session. Layered wound closure was performed without drains. Intraoperative cultures confirmed MSSA in all cases. All three surgeries were performed by one senior knee surgeon (M.S.).

### Postoperative course and outcomes

Postoperatively, all patients received 6 weeks of intravenous vancomycin, oral rifampin, and 14 days of meropenem, under the supervision of an infectious diseases specialist, transitioning to 8 weeks of outpatient oral clindamycin. Although intraoperative cultures confirmed MSSA, initial postoperative intravenous antibiotics—vancomycin and meropenem—were utilized to ensure broad coverage while awaiting sensitivities and were later tailored. Oral rifampin was added for its biofilm-penetrating properties. Once MSSA was confirmed, meropenem was discontinued after 14 days, and vancomycin was continued due to institutional protocol in high-risk patients. Anti-rheumatoid therapy in the first patient was reinitiated 6 weeks postoperatively under rheumatologic consultation. Early mobilization and physical therapy were initiated in all cases.

All three patients were monitored with serial inflammatory markers following surgery. In each case, both CRP and ESR remained elevated during the first four postoperative weeks despite clinical improvement, gradually declining thereafter. By postoperative week 8, inflammatory markers had normalized in all patients.

• Case 1: ESR declined from 92 mm/h preoperatively to 43 mm/h at 4 weeks and 12 mm/h at 8 weeks; CRP declined from 185 mg/L to 36 mg/L at 4 weeks and <5 mg/L by 8 weeks.
• Case 2: ESR decreased from 76 mm/h to 39 mm/h at 4 weeks and normalized to 14 mm/h at 8 weeks; CRP fell from 132 mg/L to 29 mg/L at 4 weeks and 6 mg/L by week 8.
• Case 3: ESR declined from 98 mm/h to 46 mm/h at 4 weeks and reached 11 mm/h at 8 weeks; CRP dropped from 201 mg/L to 41 mg/L at 4 weeks and was <4 mg/L at the final follow-up.

No postoperative aspirations were required, as all patients demonstrated steady clinical recovery with decreasing effusion, normalized range of motion, and pain resolution.

**Figure 2:**
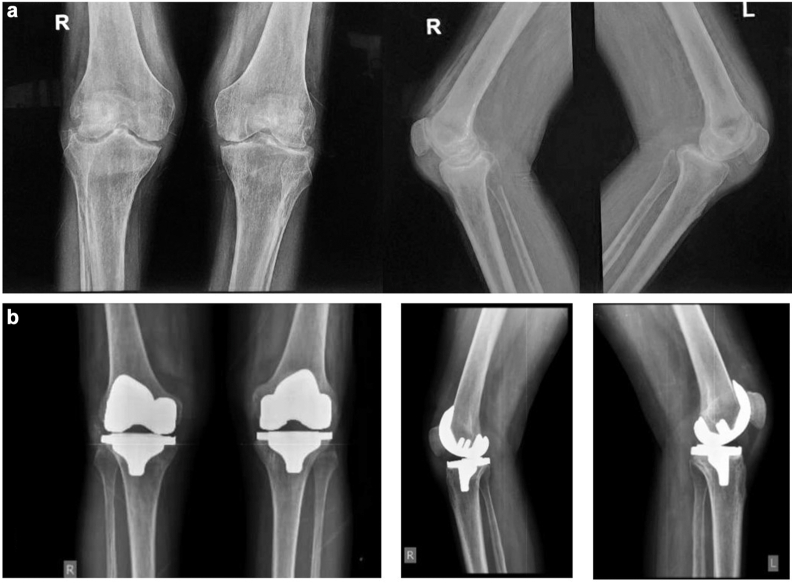
(a) Preoperative anteroposterior and lateral radiographs of both knees of case 1 demonstrating severe degenerative joint disease (DJD) with notable joint space narrowing, osteophyte formation, and significant lateral tibial plateau erosions, indicative of advanced osteoarthritic changes. (b) 24-month postoperative orthogonal radiograph of case 1 demonstrating well-fixed knee prostheses with optimal component positioning.

**Figure 3:**
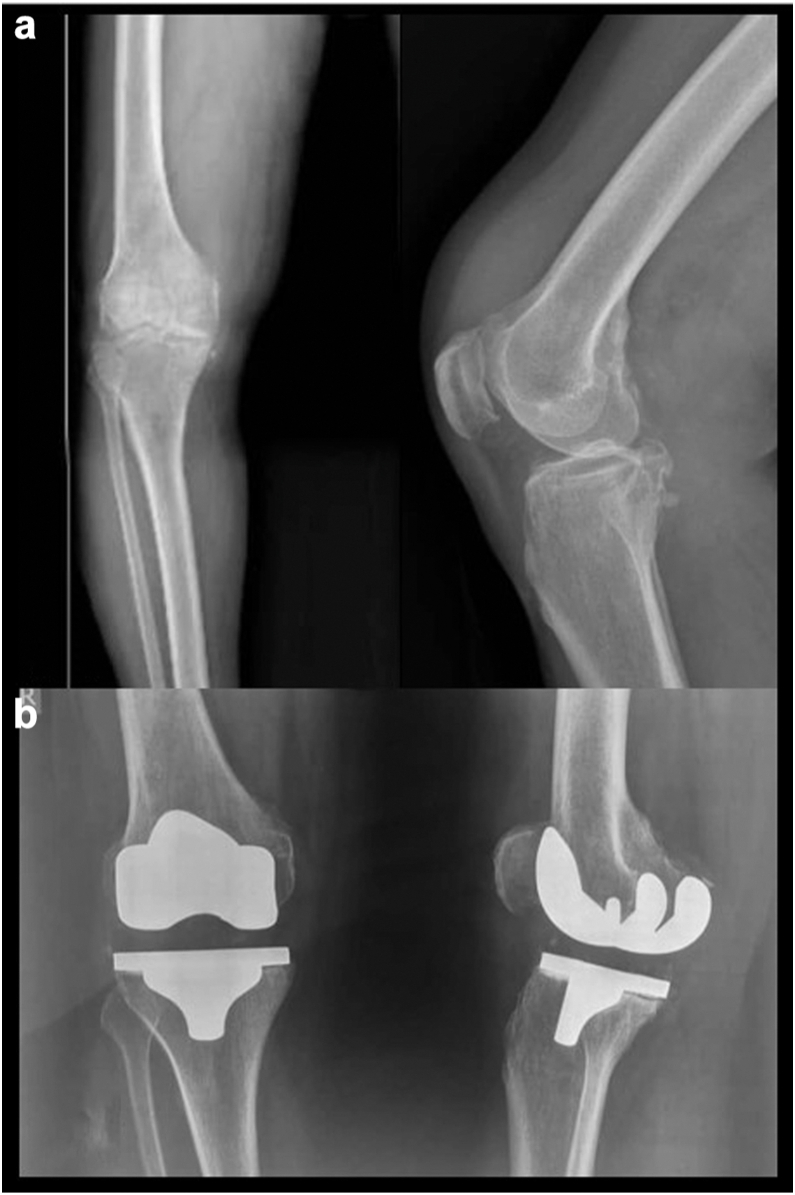
(a) Preoperative anteroposterior and lateral radiographs of the right knee in case 2, showing end-stage osteoarthritis with marked joint space narrowing, marginal osteophytes, and subchondral bone changes. (b) Postoperative anteroposterior radiograph at 24 months demonstrating stable unilateral total knee arthroplasty with well-aligned and securely fixed components.

**Figure 4:**
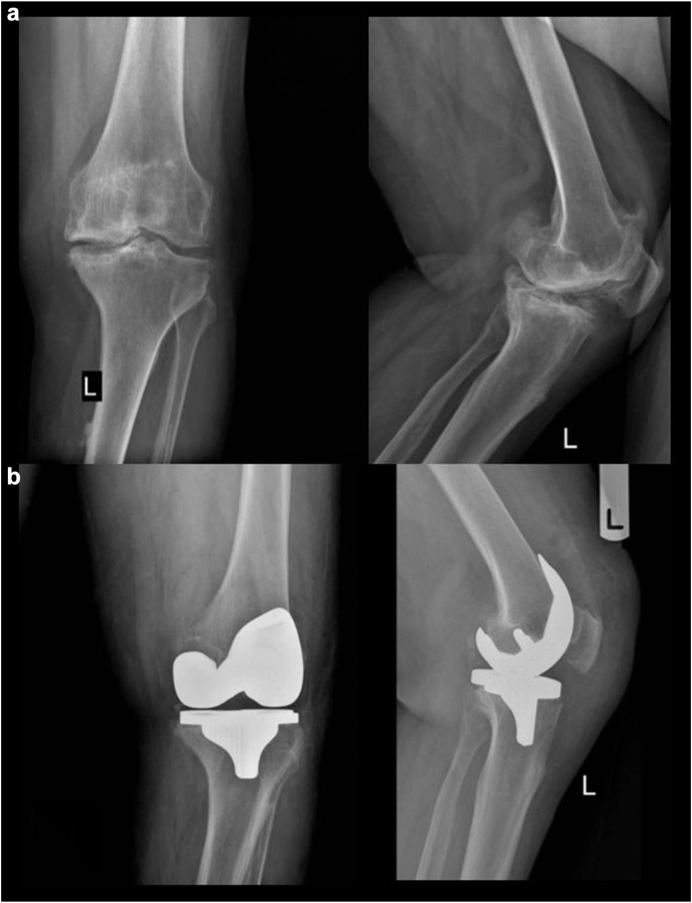
(a) Preoperative anteroposterior and lateral radiographs of the left knee in case 3 reveal advanced tricompartmental osteoarthritis characterized by pronounced joint space obliteration, marginal osteophyte formation, and subchondral sclerosis. (b) Anteroposterior and lateral radiographs obtained 24 months postoperatively demonstrate a well-positioned left total knee prosthesis with desired alignment and no signs of loosening or mechanical complications.

Serial radiographs demonstrated well-fixed prostheses without signs of loosening or periprosthetic infection at one, three, six, 12, and 24 months postoperatively (Figure 2, Figure 3, Figure 4). All patients exhibited significant functional improvement, with final follow-up outcomes as follows:

Case 1: Full extension, 100° flexion of both knees, Oxford Knee Score (OKS): 46, and Western Ontario and McMaster Universities Osteoarthritis Index (WOMAC) score: 11

Case 2: Full extension, 110° flexion, complete pain resolution, OKS: 46, and WOMAC score: 6

Case 3: Full extension, 105° flexion, and an OKS: 44 and WOMAC score: 8

No clinical or paraclinical evidence of recurrences of infection was observed at 2 years in any of the patients.

## Discussion

We present three cases of acute septic arthritis in osteoarthritic knees successfully managed with one-stage primary joint replacement, with 2-year follow-ups. The management of septic arthritis in the context of osteoarthritis is inherently complex, necessitating a delicate balance between eradicating infection and undertaking definitive surgical intervention. Conventional protocols advocate a staged approach [12], wherein initial aggressive debridement and systemic antimicrobial therapy precede delayed arthroplasty. Several case series have explored this paradigm, yielding heterogeneous functional outcomes and variable infection recurrence rates.

Farrell et al. [9] analyzed 10 patients undergoing TKA postseptic arthritis, with a mean interval of 13.8 months postinfection. Patients achieved an enhanced range of motion (mean 85°) and significant pain alleviation, with no recurrent infections. Despite these promising results, the authors advised against routine TKA in postinfectious knees due to the enduring risk of reinfection. Decades later, Bauer et al. [8] retrospectively reviewed 53 cases (31 knees, 22 hips), juxtaposing one- and two-stage procedures. The two-stage approach, with a mean 6-week interval and 3 months of antimicrobial therapy, achieved infection eradication in 87% of cases. Conversely, the one-stage procedure, performed after presumed infection resolution, demonstrated a 95% success rate, with analogous outcomes between knee and hip cohorts.

Tahmasebi et al. [13] investigated two-stage TKA in six patients with septic arthritis, reporting 100% infection eradication and marked functional enhancements. However, the two-stage strategy incurs significant drawbacks, including protracted immobilization, multiple hospitalizations, escalated costs [14], and increased morbidity in elderly patients with degenerative joint disease and systemic comorbidities. These limitations underscore the appeal of one-stage TKA, which consolidates infection eradication and joint reconstruction within a single operative session.

Of note, all three patients had received intra-articular injections—either corticosteroids, hyaluronic acid, or platelet-rich plasma—within 1 to 3 months prior to symptom onset. These were performed in outpatient rheumatology or pain clinics without image guidance. The temporal proximity of these injections to clinical presentation, in the absence of other infection sources, strongly suggests a causal relationship.

Septic arthritis following intra-articular injections is a rare but well-documented complication, with incidence rates ranging from 0.01% to 0.05% depending on the substance injected and the sterility of the technique used. Risk factors include immunosuppression, diabetes, advanced age, and RA [15]—all of which were present in our cohort to varying degrees. Corticosteroid injections, in particular, are associated with transient local immunosuppression that may predispose the joint to opportunistic bacterial invasion, especially in the setting of contaminated injection materials or breaches in aseptic technique [15,16].

Furthermore, injection-related infections can evolve subclinically, leading to chronic low-grade joint sepsis that may only become evident when joint destruction is advanced. Iatrogenic infections have also been reported in association with arthroscopy, meniscal procedures, and diagnostic aspirations—even when performed under seemingly sterile conditions [17]. These cases highlight the importance of thorough preoperative history-taking and caution when considering joint-preserving interventions in high-risk populations.

We believe the scarcity of reports on performing one-stage TKA in septic knees may stem from a pervasive apprehension regarding infection recurrence; however, the rationale for one-stage TKA in the context of acute septic arthritis is grounded in the principles of one-stage revisions of periprosthetic infections of previous TKAs. Considering thorough debridement, immediate biomechanical restoration by new prostheses implantation, and optimized antimicrobial therapy many authors reported favorable results in one-stage septic TKA revisions and this method is gaining popularity [[18], [19], [20], [21], [22]]. Matar et al. [23], in a retrospective study of 292 revision total knee arthroplasties for chronic periprosthetic joint infections (PJIs), compared single-stage (28.1%) and two-stage (71.9%) procedures. At a mean follow-up of 6.3 years, failure rates were 6.1% for single-stage and 12% for two-stage revisions, with all failures occurring within 4 years. Identified risk factors for failure included being over 80 years (OR: 5.96; *P* = .033) and the presence of a sinus (OR: 4.97; *P* = .006). Ten-year patient survivorship was comparable between the two groups (72% vs 70.5%; *P* = .517). Similarly, in a systematic review of 16 studies, Yaghmour et al. [22] reported the results of 3645 knee single-stage revision surgeries. Their review demonstrated favorable outcomes for single-stage revision in the management of infected TKA. The study concluded that single-stage rTKA is an effective alternative to two-stage revision in appropriately selected patients.

Of note, many cases of these one-stage scenarios are chronic PJIs with bone and soft tissue infectious involvement, sometimes even extending to metaphysis, while in most acute septic arthritis cases, the process is limited to synovium and cartilage.

In adapting principles from one-stage revision TKA protocols, key tenets applied include radical debridement, immediate mechanical reconstruction with cemented components, and a tailored antibiotic regimen based on culture data. Identification of the infecting organism is critical for targeted therapy, and this approach is not recommended in culture-negative infections due to the inability to customize antibiotics effectively. Our successful outcomes were partly attributed to the presence of MSSA in all cases, allowing intravenous and oral antibiotic coverage.

Although limited, the cases presented in this report illustrate the feasibility of this approach, demonstrating favorable clinical outcomes in patients with acute septic arthritis superimposed on osteoarthritic knees. Notably, all patients exhibited significant symptomatic relief, functional improvement, and no evidence of recurrent infection at follow-up, supporting the viability of one-stage TKA as a definitive treatment modality. The rationale for immediate TKA in the setting of native joint septic arthritis includes (1) definitive eradication of infection via radical debridement, (2) early restoration of joint function and mobility, and (3) avoidance of morbidity associated with staged procedures and prolonged immobilization. Although controversial, this approach aligns with principles from one-stage revision arthroplasty and may be appropriate for select patients with a known organism, controlled comorbidities, and no sinus tract. In our institution, this is not yet the standard of care for all arthroplasty surgeons, but rather a protocolized option used selectively by the lead surgeon and his arthroplasty team under infectious disease specialists' consultation.

**Figure 5:**
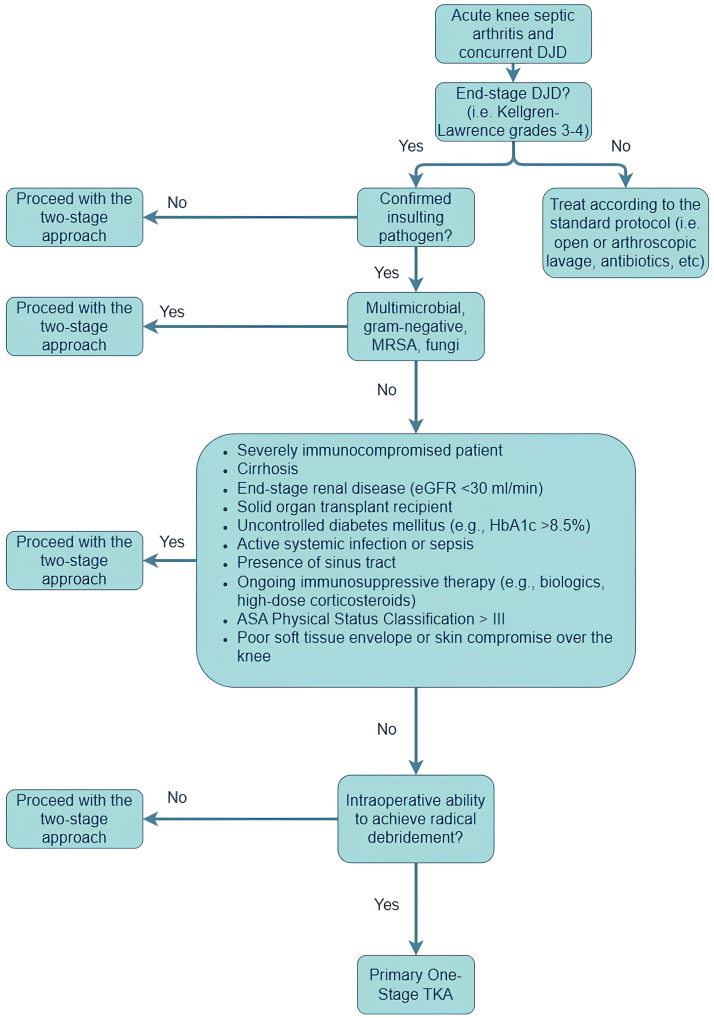
Proposed clinical decision-making algorithm for the management of acute native knee septic arthritis in the setting of concurrent degenerative joint disease. The pathway guides surgical strategy—favoring a two-stage approach in cases with resistant organisms, systemic risk factors, or poor local tissue conditions, and recommending a primary one-stage total knee arthroplasty only when radical debridement is achievable and exclusion criteria are not met. DJD: degenerative joint disease, HbA1c, hemoglobin A1c; MRSA, Methicillin-resistant Staphylococcus aureus; eGFR, estimated glomerular filtration rate.

We believe that a critical component of successful one-stage TKA in septic arthritis is rigorous patient selection. Factors such as host immune status, microbial virulence, and comorbid conditions must be meticulously evaluated to identify candidates who are most likely to benefit from this intervention. We are against the usage of this approach in cases with highly virulent germs such as Methicillin-resistant Staphylococcus aureus, polymicrobial infections, or fungal arthritis. Additionally, gram-negative bacilli have been associated with worse outcomes in PJI and may warrant exclusion from this one-stage protocol. We recommend restricting this technique to infections with gram-positive organisms, particularly MSSA, pending further evidence for broader application. Although one of our cases was a known case of RA with prolonged corticosteroid use, immunocompromised individuals and those with extensive soft tissue compromise may be at higher risk for persistent infection and may warrant a more conservative, staged approach. Moreover, patients with cirrhosis, end-stage renal disease, organ transplants, uncontrolled diabetes mellitus (eg, hemoglobin A1c >8.5%), or other severe systemic immunocompromised should be approached with caution, as these conditions are associated with impaired infection clearance and higher failure rates in similar surgical contexts. However, for select patients with controlled systemic health and localized infection amenable to aggressive debridement, one-stage TKA offers significant advantages, including reduced surgical burden, shorter hospital stays, and accelerated rehabilitation. We propose a decision-making algorithm for septic arthritis of the native degenerative knee as shown in Figure 5.

Postoperative management is another key determinant of success in one-stage TKA for septic arthritis. Prolonged systemic antibiotic therapy, typically extending for 6 weeks, is essential to ensure complete infection resolution. Serial inflammatory markers, including CRP and ESR, serve as valuable indicators of therapeutic response and potential recurrence risk. In the present cases, sustained clinical improvement, normalization of inflammatory markers, and absence of radiographic evidence of periprosthetic infection reinforced the durability of the surgical intervention.

One technical consideration in our procedures was that, unlike the two-stage approach, no additional antibiotics (eg, vancomycin) were added to the polymethyl methacrylate cement beyond the premixed tobramycin. Although vancomycin-loaded cement helps in infection eradication, as the antibiotic diffuses through the matrix, it could potentially change the mechanical properties, resulting in lower cement strength the longer it is installed [19,24]. This issue is not important in the temporary spacer but does matter in the settings of permanent prostheses. Another point is that although all procedures in this series utilized a patented extramedullary distal femoral cutting block developed by one of the authors, we emphasize that the one-stage protocol described here is not dependent on proprietary instrumentation. The essential principles—radical debridement, sterile field change, cemented implantation, and targeted antibiotic therapy—can be achieved using widely available, standard distal femoral cutting guides.

Despite these promising findings, the potential limitations of one-stage TKA in septic arthritis must be acknowledged. Concerns regarding residual bacterial biofilm, occult infection persistence, and PJI recurrence necessitate continued vigilance in patient monitoring. Additionally, long-term prosthetic survivorship data in this specific patient cohort remain limited, warranting further research and extended follow-up studies.

## Summary

The successful implementation of one-stage primary TKA for acute septic arthritis of osteoarthritic knees, as demonstrated in our cases, highlights the feasibility and efficacy of this emerging treatment paradigm. While careful patient selection, rigorous intraoperative technique, and comprehensive antimicrobial management are paramount to success, the potential benefits of a single-stage approach—reduced surgical morbidity, expedited recovery, and enhanced patient satisfaction—warrant continued exploration and broader clinical application.

## Conflicts of interest

The authors declare there are no conflicts of interest.

For full disclosure statements refer to https://doi.org/10.1016/j.artd.2025.101777.

## Informed patient consent

The author(s) confirm that written informed consent has been obtained from the involved patient(s) or if appropriate from the parent, guardian, power of attorney of the involved patient(s); and, they have given approval for this information to be published in this case report (series).

## CRediT authorship contribution statement

**Mohammad Mahdi Sarzaeem:** Project administration, Investigation, Conceptualization. **Davood Feizi:** Data curation, Supervision, Validation, Writing – review & editing. **Hamidreza Jamshidi Kouhsari:** Data curation, Writing – review & editing. **Farzad Amouzadeh Omrani:** Writing – original draft, Software, Conceptualization. **Ali Pourmojarab:** Writing – review & editing, Writing – original draft, Data curation.

## Appendix A Supplementary data

Conflict of Interest Statement for Amouzadeh Omrani

Conflict of Interest Statement for Feizi

Conflict of Interest Statement for Jamshidi Kouhsari

Conflict of Interest Statement for Pourmojarab

Conflict of Interest Statement for Sarzaeem
