# Supplementary material for: One-Stage Primary Total Knee Arthroplasty as the Treatment of Acute Septic Arthritis of the Native Osteoarthritic Knee: Report of 3 Cases and Review of Literature
Source: Arthroplast Today. 2025 Jul 28;34:101777. doi: 10.1016/j.artd.2025.101777 (PMC12320547; doi:10.1016/j.artd.2025.101777)
Supplement: Conflict of Interest Statement for Pourmojarab [file mmc4.docx]

# CONFLICT OF INTEREST STATEMENT

***American Association of Hip and Knee Surgeons***

(Adopted from the American Academy of Orthopaedic Surgeons disclosure statement)

The following form **must be filled out completely and submitted by each author (example, 6 authors, 6 forms).**

**All items require a response. If there is no relevant disclosure for a given item, enter "*None*.”**

Manuscript Title: One-Stage Primary Total Knee Arthroplasty as the Treatment of Acute Septic Arthritis of the Native Osteoarthritic Knee: Report of 3 Cases and Review of Literature

1. Royalties from a company or supplier (The following conflicts were disclosed)

None.

2. Speakers bureau/paid presentations for a company or supplier (The following conflicts were disclosed)

None.

3A. Paid employee for a company or supplier (The following conflicts were disclosed)

None.

3B. Paid consultant for a company or supplier (The following conflicts were disclosed)

None.

3C. Unpaid consultants for a company or supplier (The following conflicts were disclosed)

None.

4. Stock or stock options in a company or supplier (The following conflicts were disclosed)

None.

5. Research support from a company or supplier as a Principal Investigator (The following conflicts were disclosed)

None.

6. Other financial or material support from a company or supplier (The following conflicts were disclosed)

None.

7. Royalties, financial or material support from publishers (The following conflicts were disclosed)

None.

8. Medical/Orthopaedic publications editorial/governing board (The following conflicts were disclosed)

None.

9. Board member/committee appointments for a society (The following conflicts were disclosed)

None.

**Each author must sign AND print or type his/her name, date and submit a separate form**

In addition, one BLINDED Conflict of Interest form (no author names used) should be submitted per manuscript with all author disclosures.

Author Name (Print or Type) Author Signature Date

Ali Pourmojarab
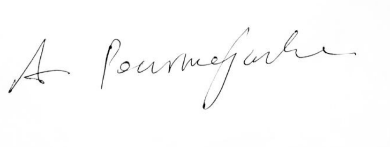
 3/4/2025
